# *wgatools*: an ultrafast toolkit for manipulating whole-genome alignments

**DOI:** 10.1093/bioinformatics/btaf132

**Published:** 2025-03-27

**Authors:** Wenjie Wei, Songtao Gui, Jian Yang, Erik Garrison, Jianbing Yan, Hai-Jun Liu

**Affiliations:** School of Life Sciences, Westlake University, Hangzhou 310030, China; National Laboratory of Crop Genetic Improvement, Huazhong Agricultural University, Wuhan 430070, China; National Key Laboratory of Wheat Improvement, College of Life Sciences, Shandong Agricultural University, Tai’an 271018, China; School of Life Sciences, Westlake University, Hangzhou 310030, China; Westlake Laboratory of Life Sciences and Biomedicine, Hangzhou 310024, China; Department of Genetics, Genomics and Informatics, University of Tennessee Health Science Center, Memphis, TN 38163, United States; National Laboratory of Crop Genetic Improvement, Huazhong Agricultural University, Wuhan 430070, China; Yazhouwan National Laboratory, Sanya 572024, China

## Abstract

**Summary:**

With the rapid development of long-read sequencing technologies, the era of individual complete genomes is approaching. We have developed *wgatools*, a cross-platform, ultrafast toolkit that supports a range of whole-genome alignment formats, offering practical tools for conversion, processing, evaluation, and visualization of alignments, thereby facilitating population-level genome analysis and advancing functional and evolutionary genomics.

**Availability and implementation:**

*wgatools* supports diverse formats and can process, filter, and statistically evaluate alignments, perform alignment-based variant calling, and visualize alignments both locally and genome-wide. Built with Rust for efficiency and safe memory usage, it ensures fast performance and can handle large datasets consisting of hundreds of genomes. *wgatools* is published as free software under the MIT open-source license, and its source code is freely available at https://github.com/wjwei-handsome/wgatools and https://zenodo.org/records/14882797.

## 1 Introduction

The advent of long-reads sequencing technologies has revolutionized genomics, enhancing the continuity and feasibility of sequencing complete genomes (Van Dijk *et al.* 2018, Li and Durbin 2024). This technological advancement is paving the way for an era where personalized genomes could become a common resource for scientific research and medical applications. Whole-genome alignment (WGA), a foundational technique in comparative genomics, plays a critical role in the analysis and interpretation of genomic data. It facilitates the identification of genetic variations and evolutionary relationships among different individuals or species. WGA techniques vary widely, each developed to address specific research needs and to optimize particular aspects of genome analysis (Dewey 2019, Kille *et al.* 2022, Song *et al.* 2024). These techniques generate data in multiple formats, such as MAF (Multiple Alignment Format, https://genome.ucsc.edu/FAQ/FAQformat.html#format5), PAF (Pairwise mApping Format, https://github.com/lh3/miniasm/blob/master/PAF.md) (Li 2018), and Chain (https://genome.ucsc.edu/goldenPath/help/chain.html), which are tailored for distinct analytical purposes (Table 1). However, the diversity of these formats poses a significant challenge: incompatibility between them impedes the seamless integration and comparison of genomic data across different studies or platforms. Consequently, researchers often find themselves confined to the data types supported by their chosen tools, which can limit the scope of their analyses and hinder collaborations.

**Table 1.** Comparison of the widely used genome alignment formats.

Format	Application scenarios	Structure	Pros	Cons	Type
Chain	Suitable for large-scale genome assembly and cross-species comparisons; Used to represent syntenic regions.	Links sets of alignment blocks that are homologous and ordered in both genomes.	Useful for long-range relationships and annotation transfer.	Lacks base-pair level detail, focusing more on structure.	Pairwise
PAF	Efficient in long-read sequencing for storing large genomic alignments.	Tab-delimited, includes basic alignment data like names, lengths, positions, and mapping quality.	Efficient with large, long-read datasets.	Omission of finer alignment details which may be crucial for certain analyses.	Pairwise
MAF	Best for comparative genomics across multiple species, phylogenetics, and evolutionary studies.	Contains blocks with alignments, each block starts with “a” and sequence lines start with “s.”	Excellent for multi-species alignments and detailed analysis.	Bulky and less efficient for very large datasets.	Multiple
Delta	Ideal for closely related genomes or small-scale differences; Used by MUMmer for basic differences between sequences.	Consists of a header and alignment blocks detailing insertions, deletions, and substitutions.	Compact and efficient for similar sequences.	Less suitable for complex rearrangements and lacks detailed visualization.	Pairwise

Recognizing these challenges, there is a critical need for a versatile, efficient tool that can bridge the gap between different genome alignment ormats, thereby facilitating a more integrated approach to genomic analysis. Such a tool could enhance data compatibility and accessibility, and also enable more comprehensive and flexible analysis, fostering collaboration and innovation in genomic research. However, there is currently no integrated software offering this capability, which prevents researchers from fully capitalizing on the wealth of information in their datasets.

**Figure 1.**
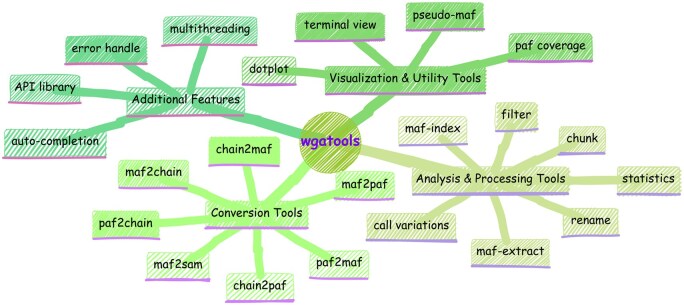
Comprehensive suite of functionalities in *wgatools*.

Here, we have developed *wgatools* to address this challenge. Programmed with Rust, *wgatools* is an ultrafast, cross-platform toolkit designed to support all major WGA formats, thereby providing efficient conversion between them. *wgatools* offers functionalities for processing, filtering, and statistically evaluating genome alignments, and includes features for variant calling and both local and genome-wide visualization (Fig. 1). The toolkit performs efficiently on standard personal computers and is robust enough to handle large-scale genomic studies involving hundreds of genomes. *wgatools* has already been used in newly developed multi-genome alignment pipeline (Zhou *et al.* 2024), in studying the evolution of complex regions that were previously uncharacterized (Yoo *et al.* 2024), and is currently being integrated into more pan-genome pipelines.

## 2 Implementation of *wgatools*

### 2.1 Format conversion

*wgatools* is equipped with a variety of tools (Fig. 1) to handle and transform genome alignment files across different formats, eliminating the need to start from scratch with specific workflows to generate particular formats. It supports conversion among three popular formats: MAF, PAF, and Chain. Our pivotal conversion step involves a set of byte-oriented, zero-copy, memory-safe, and exceptionally fast parsing combinators for the CIGAR string, an efficient compressed representation of alignment information. This ensures rapid and reliable parsing, significantly enhancing the performance and usability of our toolkit in genomic analysis.

### 2.2 Data processing and analysis

*wgatools* offers extensive data processing and analysis capabilities that significantly enhance its utility in genomic data analysis. The toolkit supports efficient indexing and precise extraction of specific intervals from MAF files, streamlining the handling of large alignment datasets. Furthermore, it allows for the segmentation of MAF files into smaller, sequential, and manageable chunks, facilitating parallel processing and subsequent analysis.

*wgatools* provides comprehensive statistical summaries and filtering for various alignment files, offering valuable insights into alignment quality and characteristics. Additionally, it supports advanced functionalities for pairwise genome alignments across multiple species, including computing coverage metrics and generating pseudo-MAF format, which enables efficient downstream comparative genomics and evolutionary studies. For example, *wgatools* has been utilized in the Ape T2T genome project to identify key genomic differences, such as single-nucleotide variants (SNVs) and structural variants (SVs), as well as conserved regions across species. Gap divergence was calculated across 1 Mb segments, revealing critical evolutionary distinctions between humans and non-human primates. Furthermore, the pseudo-MAF facilitated the generation of per-base conservation scores, which uncovered conserved regions like the major histocompatibility complex (MHC) locus and highlighted areas of rapid evolutionary change (Yoo *et al.* 2024).

By effectively managing and analyzing complex genomic data, *wgatools* could enable significant biological discoveries and deepen our understanding of genomic diversity.

### 2.3 Variant identification

*wgatools* uses efficient algorithms to identify various genomic variations by distinct alignment signatures, including SNPs, insertions, deletions, and other structural variations. By accurately detecting these mutations, researchers can gain valuable insights into the genetic diversity within and between species. The mutation identification module of *wgatools* is highly customizable, allowing users to define specific output fields and filters to tailor the analysis to their research needs.

Owing to these advantages, *wgatools* is being actively used to evaluate the efficacy of state-of-the-art genome-wide alignment software. Specifically, *wgatools* has proven valuable for assessing genome aligners, including wfmash (Guarracino *et al.* 2023), minimap2 (Li 2018), and AnchorWave (Song *et al.* 2022), by enabling direct variant calling from alignments. These calls, with or without further alignment filtering, can also be compared with long-read mapping-based calls to assess alignment accuracy. This application highlights the role of *wgatools* as a reliable benchmark for comparing the performance of various alignment tools.

### 2.4 Visualization of alignment results

Understanding complex genomic data can be greatly facilitated by effective visualization. *wgatools* provides two visualization modules to help researchers explore and interpret genomic variations intuitively:

1. **Terminal User Interface (TUI):** Given that most bioinformatics analyses are conducted through the terminal, this module is highly convenient. Users can execute commands and display results interactively using only the keyboard.
2. **Interactive Dot Plot:** This module allows users to drag and scale the field of view freely, facilitating an in-depth understanding of the genomic relationships from various perspectives. Additionally, it offers the flexibility to switch between individual-variant-level and overview-level views, enhancing the interpretability of complex genomic data.

### 2.5 Performance and usability

*wgatools* is written in Rust, a language known for its memory safety, concurrency support, and execution efficiency. This ensures robustness and efficient handling of large datasets. *wgatools* stands out for its speed. Unlike the slower, disparate personal Python scripts often used in this domain, *wgatools* is significantly faster, even when compared to similar Rust-based tools, in format conversion. For example, it achieves approximately five times faster performance than paf2chain (Guarracino 2023).

Designed for user-friendly and versatility, it offers numerous parameters, shell auto-completion, robust error management, efficient multi-threading, and supports various compressed formats. In addition to its command-line tools, *wgatools* also incorporates a comprehensive Rust library, providing developers with a powerful, low-level API for seamless integration into their software development processes and workflows. This facilitates efficient handling of genomic data and supports the development of high-performance, custom bioinformatics applications.

*wgatools* is reproducible and reliable across multiple platforms. It can be easily installed from widely distributed sources like Bioconda, Nix, Docker, and Singularity.

### 2.6 Future directions

Future development of *wgatools* will focus on supporting more efficient formats such as HAL (Hierarchical ALignment), which is essential for comparative genomics (Hickey *et al.* 2013). Additionally, *wgatools* will integrate formats related to graph-based pan-genomes, a key future direction in genomics. By supporting these advanced formats, *wgatools* aims to facilitate comprehensive and ongoing genomic analysis continuously, ensuring it remains an essential tool for addressing the challenges posed by increasingly complex genomic datasets.

## 3 Conclusions

We have presented *wgatools*, an ultrafast toolkit for manipulating WGAs, representing a significant advancement in comparative genomics data analysis. *wgatools* offers unprecedented speed and versatility, performing format conversion, data processing, statistical analysis, mutation identification, and visualization with efficiency. Its capabilities make it a valuable tool for researchers seeking to gain meaningful insights from complex genomic datasets.

*wgatools* enhances downstream analyses by integrating data from different pipelines and swiftly converting between formats. It fosters collaboration and data sharing among researchers, enabling easy comparison and combination of results obtained using different methods. This would facilitate a deeper understanding of genomic variations and their impacts in various biological contexts.

The widespread adoption of *wgatools* by numerous users highlights its utility and effectiveness, underscoring its reliability and performance as one of the leading tools for comparative genomic data analysis.

## Data Availability

There are no new data associated with this article.
